# Biaxial Tensile Strain-Induced Enhancement of Thermoelectric Efficiency of *α*-Phase Se_2_Te and SeTe_2_ Monolayers

**DOI:** 10.3390/nano12010040

**Published:** 2021-12-23

**Authors:** Shao-Bo Chen, Gang Liu, Wan-Jun Yan, Cui-E Hu, Xiang-Rong Chen, Hua-Yun Geng

**Affiliations:** 1 College of Physics, Institute of Atomic and Molecular Physics, Sichuan University, Chengdu 610064, China; shaobochen@yeah.net; 2 College of Electronic and Information Engineering, Anshun University, Anshun 561000, China; yanwanjun7817@163.com; 3 School of Physics and Engineering, Henan University of Science and Technology, Luoyang 471023, China; liugang8105@haust.edu.cn; 4 College of Physics and Electronic Engineering, Chongqing Normal University, Chongqing 400047, China; 5 National Key Laboratory for Shock Wave and Detonation Physics Research, Institute of Fluid Physics, CAEP, Mianyang 621900, China; s102genghy@caep.cn

**Keywords:** biaxial-tensile strain, *α*-phase structure, lattice thermal conductivity, thermoelectricity

## Abstract

Thermoelectric (TE) materials can convert waste heat into electrical energy, which has attracted great interest in recent years. In this paper, the effect of biaxial-tensile strain on the electronic properties, lattice thermal conductivity, and thermoelectric performance of *α*-phase Se_2_Te and SeTe_2_ monolayers are calculated based on density-functional theory and the semiclassical Boltzmann theory. The calculated results show that the tensile strain reduces the bandgap because the bond length between atoms enlarges. Moreover, the tensile strain strengthens the scatting rate while it weakens the group velocity and softens the phonon model, leading to lower lattice thermal conductivity *k*_l_. Simultaneously, combined with the weakened *k*_l_, the tensile strain can also effectively modulate the electronic transport coefficients, such as the electronic conductivity, Seebeck coefficient, and electronic thermal conductivity, to greatly enhance the *ZT* value. In particular, the maximum n-type doping *ZT* under 1% and 3% strain increases up to six and five times higher than the corresponding *ZT* without strain for the Se_2_Te and SeTe_2_ monolayers, respectively. Our calculations indicated that the tensile strain can effectively enhance the thermoelectric efficiency of Se_2_Te and SeTe_2_ monolayers and they have great potential as TE materials.

## 1. Introduction

Thermoelectric materials have drawn considerable attention because they can harvest energy from waste heat by converting thermal energy directly into electrical energy [1,2,3]. The conversion efficiency of a TE material can be evaluated by the dimensionless figure of merit, $ZT=S^{2}\sigma T/(k_{e}+k_{l})$, where *S*, *σ*, *T* are the Seebeck coefficient, the electrical conductivity, and the absolute temperature, respectively. *k*_e_ and *k*_l_ are electronic and lattice thermal conductivities. High thermoelectric performance requires a large thermoelectric power factor ($\mathrm{PF}=S^{2}\sigma$) and low thermal conductivity. However, the intrinsic relationship among these crucial parameters makes it difficult to improve the *ZT* value of a TE material.

In 2017, Zhu et al. [4] and Chen et al. [5] predicted and successfully synthesized the tellurene on highly oriented pyrolytic graphite (HOPG) substrates by using molecular beam epitaxy. Subsequently, a new two-dimensional (2D) materials family, the group-VI elemental 2D materials, has attracted significant attention due to its high carrier mobility, high photoconductivity, and thermoelectric responses [6,7,8,9,10,11]. Recent studies confirmed that the compounds composed of Te and Se have excellent thermoelectric and electronic transport properties [7,8,12]. In our previous work [7], we revealed that the 1T-phase Se_2_Te and SeTe_2_ monolayers are promising medium-temperature thermoelectric materials; however, their room temperature conversion efficiency is inferior. Recently, numerous studies [13,14,15,16] proved that the tensile mechanic strain can induce the reduction of the lattice thermal conductivity and then enhancement of the thermoelectric performance of 2D materials. Therefore, expecting to enhance the low-temperature thermoelectric efficiency, we here investigate the effect of biaxial tensile strain on the properties of *α*-phase Se_2_Te and SeTe_2_ monolayers.

In this paper, the small biaxial-tensile strain effects on electronic properties, lattice thermal conductivity, and thermoelectric performance of *α*-phase Se_2_Te and SeTe_2_ monolayers are calculated by first-principles calculations combined with the semiclassical Boltzmann theory. The calculated results indicate that the tensile strain results in lower lattice thermal conductivity by strengthening the scatting rate while at the same time weakening the group velocity and softening the phonon model. Additionally, the *ZT* value is visibly enlarged upon applied tensile strain; for example, the maximum n-type doping *ZT* under 1% and 3% strain increases up to six and five times higher than the corresponding *ZT* without strain for the Se_2_Te and SeTe_2_ monolayers, respectively. Our calculations confirm that the tensile strain is an effective way to enhance the thermoelectric efficiency of Se_2_Te and SeTe_2_ monolayers, which can stimulate further experimental works.

## 2. Theoretical Methods and Computational Details

All calculations are based on the first-principles calculations implemented in the Vienna ab initio Simulation Package (VASP) code [17,18] in the framework of the density functional theory (DFT). To solve the Kohn–Sham equations, the generalized gradient approximation (GGA) within the Perdew–Burke–Ernzerhof (PBE) formulation [19] or the B3LYP(B3PW) [20,21] is widely used to describe exchange-correlation potential. In this study, we used the former method to describe the exchange-correlation potential. A plane wave cutoff was set to 500 eV, and dense k-meshes of 14 × 14 × 1 and 22 × 22 × 1 were used to sample the Brillouin zone for structure optimizations and electronic property calculations, respectively. A vacuum space larger than 15 Å was used to avoid interactions of the nearest layers. The structures were fully optimized until the convergence threshold for electronic and ionic relaxations reached 10^−8^ eV and 10^−3^ eV/Å, respectively. The spin-orbit coupling (SOC) was taken into account in all calculations of the electronic properties.

Recently, Yang’s group developed a new TransOpt code [22] for calculating the electron transport properties based on semi-empirical Boltzmann equation with a constant electron–phonon coupling approximation. The advantage of TransOpt is not only more accurate than the constant relaxation time approximation (CRTA) but also can effectively avoid band crossing problems. Numerous studies [22,23,24,25,26] demonstrated that TransOpt is reasonable for calculating the transport properties and can be used in high-throughput calculations. In general, the Seebeck coefficient *S* and electrical conductivity *σ* are evaluated by the formula as followed [27,28,29,30]

(1) $$S\left(\mu ,T\right)=\frac{ek_{B}}{\sigma }\int d\varepsilon \left(-\frac{\partial f_{\mu }\left(T,\varepsilon \right)}{\partial \varepsilon }\right)\Xi \left(\varepsilon \right)\frac{\varepsilon -\mu }{k_{B}T}$$

(2) $$\sigma \left(\mu ,T\right)=e^{2}\int d\varepsilon \left(-\frac{\partial f_{\mu }\left(T,\varepsilon \right)}{\partial \varepsilon }\right)\Xi \left(\varepsilon \right)$$

where $f_{\mu }\left(T,\varepsilon \right)$, *k*_B_, *e*, and *ε* are the Fermi–Dirac distribution function, Boltzmann constant, electric charge, and band energy, respectively. The transport distribution is derived as $\Xi \left(\varepsilon \right)=\sum_{k}v_{k}⊗v_{k}\tau_{k}$, where $v_{k}$ and $\tau_{k}$ are the group velocity and relaxation time at state k, respectively. $\tau_{k}$ is a vital parameter to accurately calculate σ under the CRTA, which depends on the scattering mechanisms including phonon, impurity, and defect scatterings. If only the intrinsic electron–phonon scatterings are considered, the relaxation time can be defined as [22,31,32]

(3) $$\begin{array}{cc} \frac{1}{\tau_{nk}}=\frac{2\pi }{\hbar } & \sum_{mk’\lambda }|g_{mk’,nk}^{\lambda }{|}^{2}\left\{\left[f_{mk’}+n_{q\lambda }\right]\delta \left(\varepsilon_{mk},-\varepsilon_{nk}-\hbar \omega_{q\lambda }\right)\delta_{k+q,k},\right.\\ \; & \left.+\left[1+n_{q\lambda }-f_{mk}\right]\delta \left(\varepsilon_{mk},-\varepsilon_{nk}+\hbar \omega_{q\lambda }\right){\delta ’}_{k-q,k}\right\}, \end{array}$$

where $\left|g_{mk^{’},nk}^{\lambda }\right|$ describes the electron–phonon coupling matrix; *f*_*mk*’__(_f_*mk*__)_ is the Fermi–Dirac distribution for band-index m and wave-vector K; $\delta \left(\varepsilon_{mk},-\varepsilon_{nk}-\hbar \omega_{q\lambda }\right)$ and $\delta \left(\varepsilon_{mk},-\varepsilon_{nk}+\hbar \omega_{q\lambda }\right)$ expresses the absorption and emission of a phonon *ω*_qλ_, respectively; *n*_qλ_ denotes the phonon number under the Bose–Einstein distribution.

The harmonic interatomic force constants (IFCs) were obtained by the PHONOPY code [33] based on the density functional perturbation theory (DFPT) using a 5 × 5 × 1 (3 × 3 × 1) supercell and a 6 × 6 × 1 (3 × 3 × 1) k-mesh for both strained and unstrained Se_2_Te (SeTe_2_) monolayers. We then obtained the phonon frequencies by diagonalized dynamic matrix. The finite displacement approach [33,34] was performed to calculate the IFCs with a 4 × 4 × 1 and a 3 × 3 × 1 supercell for Se_2_Te and SeTe_2_ monolayers, respectively, considering the third nearest neighbors. Combining with the above calculated harmonic and anharmonic IFCs, the lattice thermal conductivity was evaluated using the ShengBTE code [35] by iterative solution of the phonon Boltzmann transport equation. After the lattice thermal conductivity convergence test, very dense q-meshes of 160 × 160 × 1 and a scalebroad of 0.7 were employed in all calculations.

## 3. Results and Discussion

### 3.1. Stability and Electronic Properties

**Figure 1:**
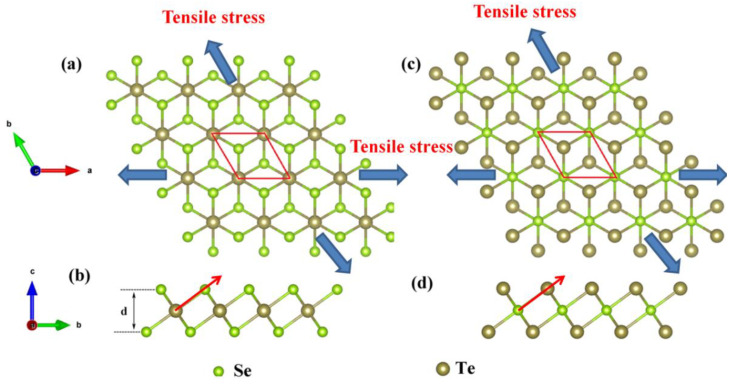
(Color online) Structure diagrams of α-phase Se2Te and SeTe2 monolayers. The (**a**) top and (**b**) side view of the Se2Te monolayers, and (**c**) top and (**d**) side view of the SeTe2 monolayers. The blue arrow indicates the direction of tensile stress. The solid red diamond-shaped box represents the primitive cell. The red arrow demonstrates the direction of the ELF profile.

The top and side views of the *α*-phase Se_2_Te and SeTe_2_ monolayers are shown in Figure 1. The same structure as 1T phase MoS_2_, Se_2_Te and SeTe_2_ monolayers belong to the P-3m1 space group and C_3v_ point group symmetry. After full relaxation, the lattice parameters are a = b = 3.98 Å, and a = b = 4.02 Å for Se_2_Te and SeTe_2_ monolayers, respectively, agreeing well with the previous report [36]. To investigate the influence of tensile strain on materials properties, the in-plane biaxial tensile strain raised from biaxial tensile stress was considered, as depicted in Figure 1. The biaxial tensile strain is defined as, $\varepsilon =\left(a-a_{0}\right)/a_{0}\times 100\%$, where *a* and *a*_0_ are the in-plane lattice parameter of the strained and unstrained monolayers, respectively.

**Figure 2:**
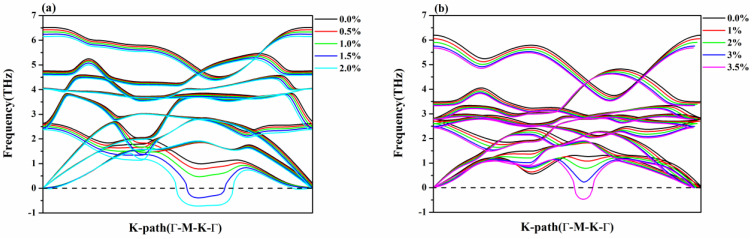
(Color online) Phonon dispersion curves under different tensile strains for (**a**) Se_2_Te and (**b**) SeTe_2_ monolayers.

Generally speaking, it is vital to check the stability of materials before calculating the properties. Thus, the phonon spectrums of Se_2_Te and SeTe_2_ monolayers under unstrained and strained structures were investigated by PHONOPY code, as shown in Figure 2. It is found that there is no imaginary frequency in the Brillouin zone at the range of given tensile strain, such as 0–3% strain for SeTe_2_ and 0–1% strain for Se_2_Te, suggesting that they are dynamic stability within a small tensile strain. Furthermore, tensile strain softens phonon mode, which may enhance the thermoelectric performance [13]. Overall, the longitudinal acoustic (LA) and transverse acoustic (TA) branches are linear near the Γ point, while the out-of-plane acoustic (ZA) branch is quadratic near the Γ point, and all of them shift to lower frequency upon the tensile strain.

**Figure 3:**
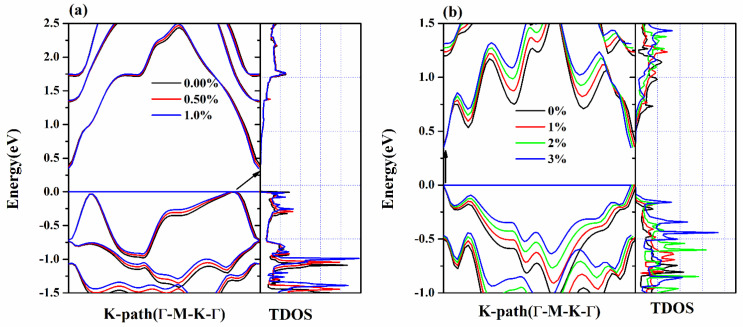
(Color online) Band structures and TDOS of (**a**) Se_2_Te and (**b**) SeTe_2_ under various biaxial tensile strains.

It is vital to correctly calculate the electronic band structure and total density of states (TDOS) related to the thermoelectric properties of a semiconductor [37,38]. It is well known that the SOC effect [39] plays a crucial role in the electronic band structure of materials containing heavy elements such as Te, thus the SOC effect is taken into account in the calculations. As depicted in Figure 3, the electric band structures and TDOS of unstrained and strained structures are calculated at the PBE + SOC level. For both Se_2_Te and SeTe_2_ monolayers, it is found that the HOMO energy reduces with the tensile strains, while the LUMO energy enhances, leading to narrowing of the bandgap. Moreover, in Table 1, the influence of tensile strain on the bandgap of Se_2_Te is significantly greater than that of SeTe_2_. For example, the bandgap of Se_2_Te is reduced by 11.11% under the effect of 1% tensile strain, while the bandgap of SeTe_2_ is reduced slightly by 3.79% under 3% tensile strain. This reduction of bandgap induced by strain results from the well-known rule that tensile strain increases the bond length between atoms, which leads to a decrease in the bandgap [14,40,41]. Furthermore, it is found that the TDOS of Se_2_Te and SeTe_2_ monolayer under the Fermi level increases and shifts up as tensile strain increases, while the TDOS above the Fermi level remains almost the same in Se_2_Te monolayer and decreases in the SeTe_2_ monolayer.

**Figure 4:**
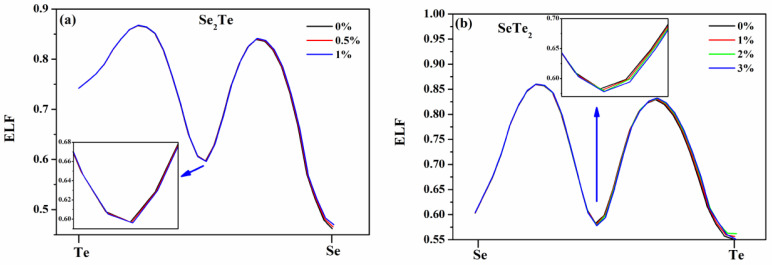
(Color online) The ELF profile from (**a**) Te to Se atom in the Se_2_Te monolayers and (**b**) Se to Te atom in the SeTe_2_ monolayers as indicated by the red arrow in Figure 1b,d, respectively. The inset shows the ELF value of the middle region of the two atoms.

Generally, the electron localization function (ELF) [42] can be used to determine the interaction type (chemical bonding type or physical binding type) between two atoms by measuring electron localization in the atomic and molecular systems [42,43]. The ELF is a relative measurement of the electron localization, and it takes values between 0 and 1 [44], where 1 corresponds to perfect localization, 0.5 means electron-gas-like pair probability, and 0 represents the absence of electrons. In other words, due to the lack of electrons sharing in the region between the two atoms, the value of ELF is very low, which represents the ionic binding; on the contrary, due to the abundant of electrons sharing in the region between the two atoms, the value of ELF is large, and it characterizes a covalent bond. To gain further insight into the effect of tensile strain on the character of the bond, we calculated ELF of Se_2_Te and SeTe_2_ monolayers, as shown in Figure 4. Overall, it is found that all ELF are larger than 0.5 at tensile-strained circumstances, indicating that they are covalent bond compounds. Furthermore, the values of ELF decrease with the increasing strain, due to the increase in the length of chemical bonds between two atoms.

### 3.2. Electronic Transport Property

We then evaluated the electrical conductivity *σ*, Seebeck coefficient *S*, electronic thermal conductivity *k*_e_, and thermoelectric power factor PF using the TransOpt package [22]. Deformation potential (DP) *E*_l_, Young’s modulus *Y*, and Fermi energy are the main input parameters for electronic transport performances, as shown in Table 1. The finite-difference method [45,46,47,48,49] was used to calculate the Young’s modulus Y. $E_{l}$^VBM^ and $E_{l}$^CBM^ are the deformation potential of the VBM and CBM in the transport direction, respectively, which can be calculated by the formula: $E_{l}=\frac{\partial E_{edge}}{\partial (\Delta l/l_{0})}$. Eedge is the energy of the VBM or CBM under slight uniaxial strain, ranging from −2% to 2% in steps of 0.5%. The elastic modulus *C*_2D_ is evaluated by the formula: $C_{2D}=\frac{1}{S_{0}}\frac{\partial^{2}E}{\partial {\left(\Delta l/l_{0}\right)}^{2}}$, where *E* is the total energy of materials under slight strain. The calculated *C*_2D_ agrees well with the results of Reference [36], indicating that the calculated results are reliable. As shown in Figure S1 (Supplemental Materials), the DP coefficient *E*_l_ is obtained by fitting the values of the band energies of the VBM and the CBM concerning the vacuum energy as a function of uniaxial strain. To reach convergence and obtain accurate calculation results, we used dense k-meshes of 60 × 60 × 1.

**Figure 5:**
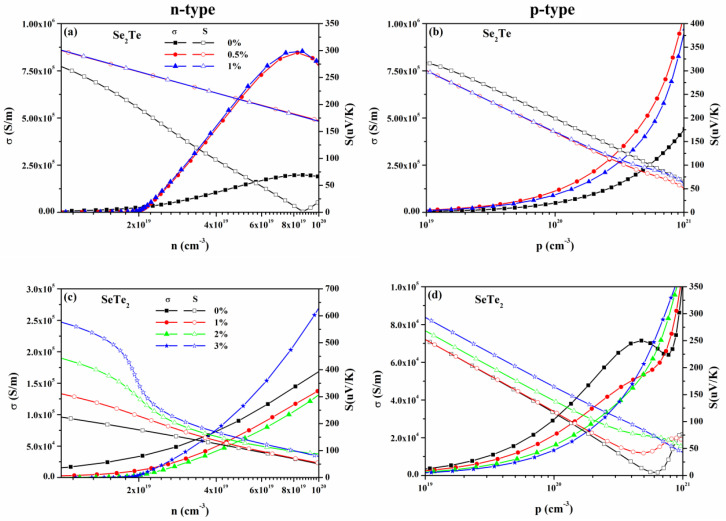
(Color online) The electronic conductivity *σ* and Seebeck coefficient *S* of (**a**,**b**) Se_2_Te and (**c**,**d**) SeTe_2_ monolayers with different applied strain as a function of concentration for both n- and p-type doping at 300 K.

**Figure 6:**
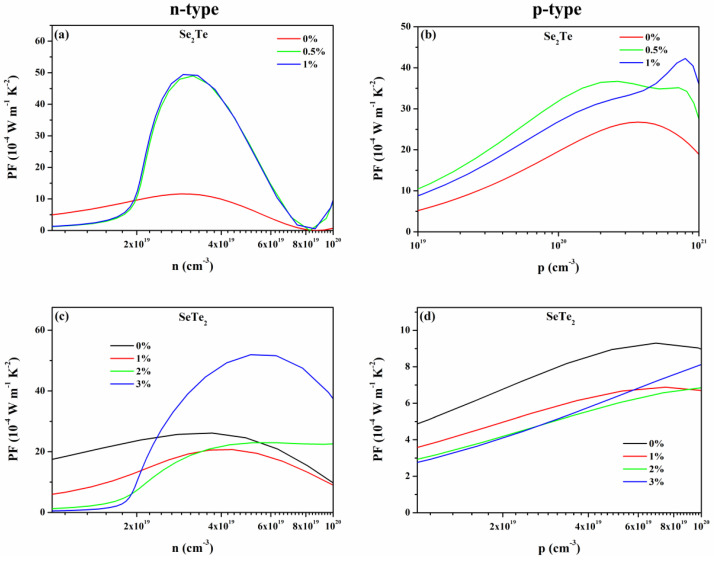
(Color online) The thermoelectric power factor PF of (**a**,**b**) Se_2_Te and (**c**,**d**) SeTe_2_ monolayers under tensile strains at 300 K as a function of concentration, respectively.

From Figure 5, at given carrier concentration range, such as 10^19^ to 10^20^ cm^−3^ and 10^19^ to 10^21^ cm^−3^ for n- and p-type doping, respectively, all types of *S* approximately linear decreases with increasing carrier concentration, while σ increases with carrier concentration. This can also be found in many other 2D materials that *S* and *σ* have a different relationship to carrier concentration since they have an opposite dependency with the DOS near the Fermi surface [7,37,50]. Thus, we can obtain the optimized PF shown in Figure 6 by the relationship of $\mathrm{PF}=S^{2}\sigma$, which is a vital parameter for the figure of merit and is discussed in the following. Moreover, except for p-type doped *S* of Se_2_Te, strain enhances the *S* as the increasing strain. Loosely speaking, the strain induces an enhancement of *σ* for Se_2_Te, while it works oppositely for that of SeTe_2_.

### 3.3. Lattice Thermal Conductivity

**Figure 7:**
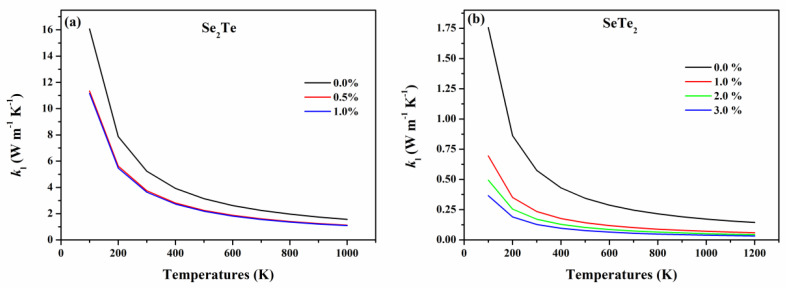
Strain-dependent lattice thermal conductivity *k*_l_ of (**a**) Se_2_Te and (**b**) SeTe_2_ monolayers as a function of temperatures.

By comparison, the *k*_l_ of SeTe_2_ monolayers is much lower than that of Se_2_Te monolayers, as shown in Figure 7, which is benefited from the large weight of the constituent elements [10] and weak bonding [51,52] (see Figure 4). Notably, it can be seen from Figure 7 that both the *k*_l_ of Se_2_Te and SeTe_2_ decrease with the biaxial tensile strain. This may result from phonon softening [13].

**Figure 8:**
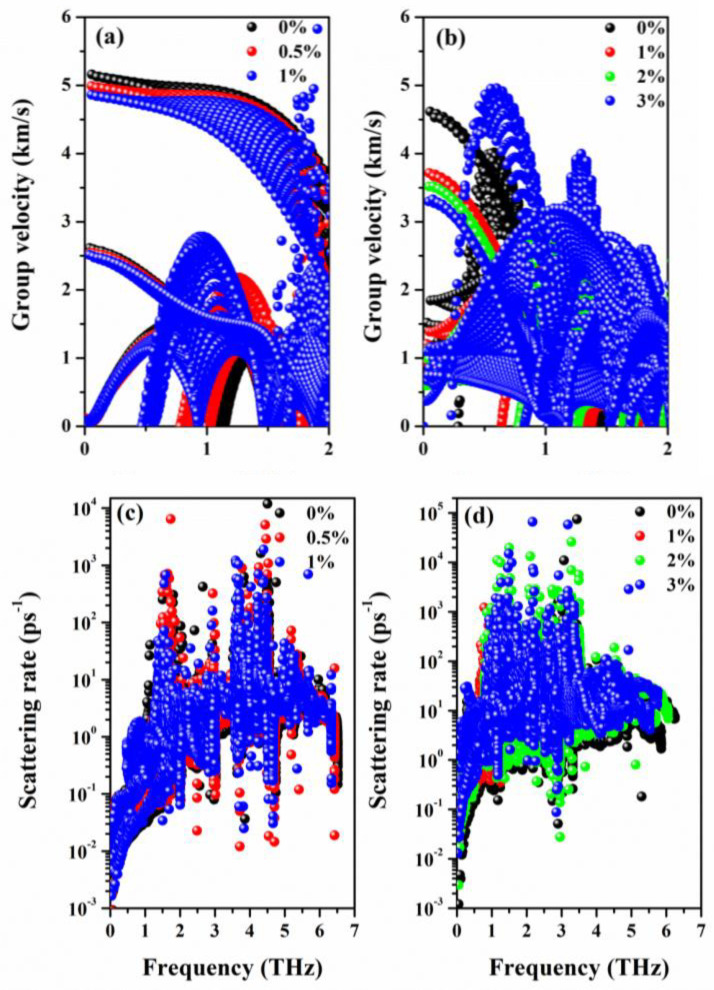
(Color online) The tensile-strained phonon group velocity of (**a**) Se_2_Te and (**b**) SeTe_2_ monolayers. Phonon scattering rate of (**c**) Se_2_Te and (**d**) SeTe_2_ under applied tensile strain.

To further understand this reduction in *k*_l_ subjected to strain, we analyzed the effect of strain on phonon group velocities and phonon scattering rates as plotted in Figure 8. According to the calculated cumulative lattice thermal conductivity shown in Figure S2, we confirm that both Se_2_Te and SeTe_2_ follow the conventional criteria that the lattice thermal conductivity is primarily carried by a low-frequency phonon, thus the phonon group velocity in the low-frequency range (0~2 THz) is plotted. Compared to Se_2_Te, the scattering rate is superior in SeTe_2_ monolayers under unstrained and strained structures, while the opposite pattern appears in the low-frequency phonon group velocity, particularly at the long-wavelength limit. Thus, the SeTe_2_ monolayers possess lower *k*_l_ compared to that of the Se_2_Te monolayers [53]. Loosely speaking, for both Se_2_Te and SeTe_2_ monolayers, the tensile strain has opposite effects on the scattering rates and phonon group velocity, that is, the tensile strains generally increase the scatting rate while the group velocity is weakened by the tensile strain. As a result, the tensile strain distinctly reduces the value of *k*_l_. Furthermore, at room temperature, the *k*_l_ of Se_2_Te and SeTe_2_ monolayers reduced by 35.5% and 77.7% under 1% and 3% tensile strain, as summarized in Table 2, respectively. Hence, suitable tensile strain is favorable for thermoelectric performance by the reduction of the *k*_l_, which is a very effective method to achieve enhanced *ZT*. Finally, by fitting the temperature-dependent *k*_l_, it is found that *k*_l_ fulfills T^−1^ behavior in all circumstances, indicating a dominant Umklapp process of phonon scattering that causes thermal resistivity [11,50].

### 3.4. Figure of Merit

**Figure 9:**
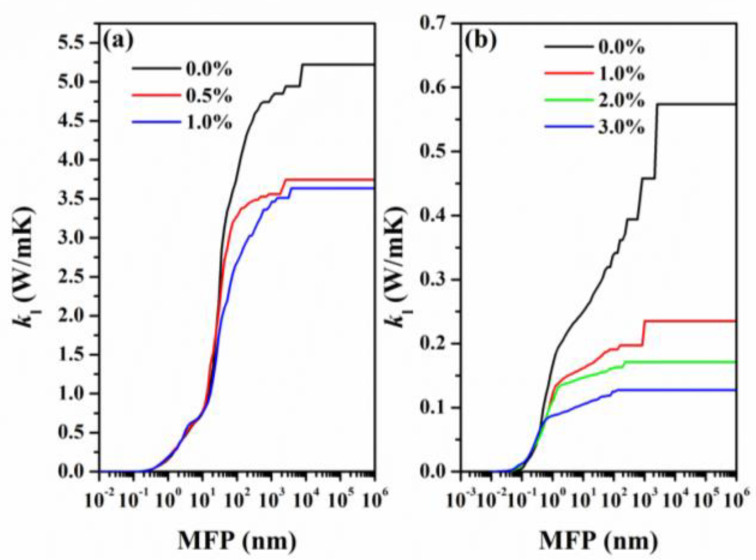
(Color online) The cumulative lattice thermal conductivity of (**a**) Se_2_Te and (**b**) SeTe_2_ monolayers under tensile strains as a function of the phonon mean free path (MFP) at room temperature.

To evaluate the size effect on the ballistic or diffusive phonon transport, we calculated the maximum phonon mean free path (MFP) distribution as plotted in Figure 9, which is important for thermal design with nanostructuring. In particular, by referring to the relationship between MFP and *k*_l_, we can get better thermoelectric performance in the application of thermoelectric materials by effectively modulating *k*_l_. To this end, at room temperature, the cumulative thermal conductivities *k*_l_ of the Se_2_Te and SeTe_2_ monolayers as a function of the MFP under various biaxial strains are fitted to a single parametric function [35]:

(4) $$k_{l}\left(\Lambda \leq \Lambda_{max}\right)=\frac{k_{l,max}}{1+\frac{\Lambda_{0}}{\Lambda_{max}}}$$

where *k*_l,max_ is the maximum lattice thermal conductivity and $\Lambda_{max}\;$ is the cutoff MFP. By fitting the cumulative thermal conductivity *k*_l_, the phonon MFPs ${\;\Lambda }_{0}$ of the Se_2_Te and SeTe_2_ monolayers under different strains are obtained. The corresponding values are 39.70, 24.18, and 34.83 nm for Se_2_Te monolayers and 12.35, 1.29, 0.70, and 0.53 nm for SeTe_2_ monolayers, respectively, which are much smaller than those of other 2D materials [54,55]. This indicates that *k*_l_ will significantly decrease when the size of the sample for Se_2_Te and SeTe_2_ monolayers is below forty and twenty nanometers, respectively. Thus, our calculations provide an important reference for the subsequent material design. Notably, it found that the phonon MFPs decrease with increasing strains due to the integral decrease of MFP and higher contributions to *k*_l_ coming from the optical phonon branches [56], as summarized in Table 2.

We then thoroughly studied the phonon mode contributions toward the *k*_l_ at 300 K from the acoustic phonon branches (ZA, TA, and LA) and optical branches (OP), as expressed in Table 2. By comparison, the phonon mode contributions to the *k*_l_ of unstrained Se_2_Te monolayers are consistent with the report [8], and the value of *k*_l_ is also in agreement with that of the report. Moreover, it can be noticed that the *k*_l_ is mainly dominated by acoustic branches in all cases. One can easily see that the optical branches’ contributions to the *k*_l_ increase with increasing biaxial strain, and at the same time the contributions of the acoustic phonon branches decrease.

**Figure 10:**
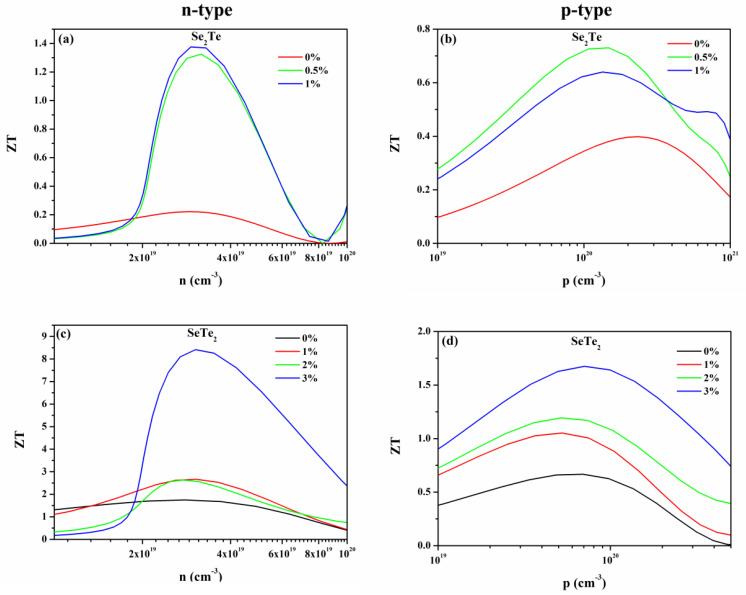
(Color online) The strain-dependent figure of merit *ZT* of (**a**,**b**) Se_2_Te and (**c**,**d**) SeTe_2_ monolayers at room temperature as a function of concentration, respectively.

To uncover the influence of biaxial strain on the thermoelectric conversion efficiency, we calculated the strain-dependent figure of merit *ZT* at T = 300 K as a function of concentration, as shown in Figure 10. For unstrained structure, the p-type doping *ZT* of Se_2_Te monolayers is superior to that of n-type doping, which is the opposite of that of SeTe_2_ monolayers. The reason for this discrepancy is interpreted in detail in our previous work [7]. For strained structures, overall, the value of n-type and p-type *ZT* was visibly enhanced for both Se_2_Te and SeTe_2_ monolayers compared to that of the unstrained structures. Particularly, the maximum n-type doping *ZT* increases up to 1.38 (8.41) under 1% (3%) strain for the Se_2_Te (SeTe_2_) monolayers, and this value is six (five) times higher than the corresponding *ZT* without strain. The corresponding maximum p-type doping *ZT* reaches up to 0.64 (1.67), which is 1.6 (2.5) times higher than that of unstrained structures. Such a high value of *ZT* of strained structures is larger than those of most reported 2D materials, such as α-Te [57], SiTe_2_ [58], SnTe_2_ [58], and XSe (X = Ge, Sn, and Pb) [50], and even larger than typically single and polycrystalline crystal SnSe [59,60], indicating tensile strain is an effective category to enhance the thermoelectric effect.

Moreover, it is also found that, except for n-type doped Se_2_Te and p-type doped SeTe_2_, the value of *ZT* does not increase monotonously as strain increases. Unlike the SeTe_2_ monolayers, the type of relatively large *ZT* value of the Se_2_Te monolayers has changed with applied strain, i.e., for the strained structure, the n-type *ZT* is greater than the p-type *ZT*, which is exactly the opposite of the unstrained structure. To reveal those phenomena and the reason how strain greatly enhanced thermoelectric performance, we calculated the PF and *k*_e_, as plotted in Figure 6 and Figure S3, respectively, due to PF and *k*_e_ being vital parameters according to the formula $ZT=PF∙T/(k_{e}+k_{l})$. Combining with the previously calculated *k*_l_, except for the p-type doped SeTe_2_ monolayers, the enhancement of thermoelectric performance induced by the strain is attributed to the simultaneous increase of the PF and decrease of the *k*_l_, which has also been found in other reports [13,61]. Although p-type PF decrease compared to the unstrained SeTe_2_ monolayers, the thermal conductivity decreases and it dominates, resulting in an enhancement of *ZT*. An interesting observation in our calculations shows that, for p-type Se_2_Te and n-type SeTe_2_, the value of *ZT* does not increase monotonously with strain, which is a result of the complicated change of PF, *k*_e_, and *k*_l_.

## 4. Conclusions

We evaluated the influence of biaxial-tensile strain on the stability, electronic properties, lattice thermal conductivity, and thermoelectric performance of α-phase Se_2_Te and SeTe_2_ monolayers by first-principle calculations combined with the semiclassical Boltzmann theory. It is found that small tensile strain softens the phonon model and reduces the phonon frequency, at the same time, the tensile strain strengthens the scatting rate and weakens the group velocity, resulting in a reduction of the lattice thermal conductivity *k*_l_. The tensile strain increases the bond length, which leads to a decrease in the bandgap. Furthermore, simultaneously combined with the weakened *k*_l_, the tensile strain can also effectively modulate the electronic transport coefficients, such as electronic conductivity, Seebeck coefficient, and electronic thermal conductivity, to greatly enhance the value of *ZT*. Our calculations indicated tensile strain can effectively enhance the thermoelectric performance of Se_2_Te and SeTe_2_ monolayers.

## Figures and Tables

**Table 1:** Deformation potential constants (*E*_l_) of α-phase SeTe_2_ and Se_2_Te based on PBE + SOC band structures under various tensile strains, together with the Young’s modulus Y, elastic modulus *C*_2D_, and bandgap E_g_, respectively.

	Strain	*E*_l_^VBM^ (eV)	*E*_l_^CBM^ (eV)	*Y* (GPa)	*C_2D_* (N/m)	E_g_ (eV)
Se_2_Te	0%	−5.086	−6.744	126.545	44.016	0.395
		−4.196 ^a^	−6.676 ^a^		43.64 ^a^	0.38 ^a^
	0.5%	−4.834	−6.552	123.247	42.547	0.373
	1%	−5.011	−6.512	120.317	41.178	0.351
SeTe_2_	0%	−6.316	−6.446	103.340	37.538	0.363
		6.590 ^a^	6.620 ^a^		36.19 ^a^	0.33 ^a^
	1%	−6.098	−6.272	97.101	34.847	0.359
	2%	−5.782	−5.956	91.257	31.590	0.354
	3%	−5.568	−5.716	85.594	30.380	0.350

^a^ Ref. [36]. *C*_2D_ was calculated using the finite-difference method by Liu et al. [36].

**Table 2:** Contributions of phonon modes (ZA, TA, LA, and all-optical) to total lattice thermal conductivity at 300 K, together with the lattice thermal conductivity *k*_l_.

Compounds	Strain	ZA	TA	LA	Optical	*k*_l_ (W m^−1^ K^−1^)
Se_2_Te	0%	17.73% 19.28% [8]	51.32% 43.12% [8]	23.65% 28% [8]	7.30% 9.29% [8]	5.236 4.88 [8]
	0.5%	18.28%	36.63%	35.50%	9.59%	3.744
	1%	21.90%	56.45%	14.14%	7.52%	3.636
SeTe_2_	0%	11.49%	6.33%	65.42%	16.76%	0.574
	1%	14.98%	10.59%	48.23%	26.20%	0.235
	2%	12.96%	17.96%	37.62%	31.46%	0.171
	3%	14.74%	20.54%	32.85%	31.86%	0.128

## Data Availability

Not applicable.

## Supplementary Materials

The following are available online at https://www.mdpi.com/article/10.3390/nano12010040/s1, Figure S1: The strain-dependent deformation potential constants *E*_l_ of *α*-phase (a-f) Se_2_Te and (g-n) SeTe_2_ monolayer based on PBE + SOC band structures, respectively. The red straight line is the linear fitting curve; Figure S2: The strain-dependent cumulative *k*_l_ of (a) Se_2_Te and (b) SeTe_2_ monolayer at 300 K as a function of frequency; Figure S3: The strain-dependent electronic thermal conductivity *k*_e_ of (a, b) Se_2_Te and (c, d) SeTe_2_ monolayer at 300 K as a function of concentration for n-type and p-type doping, respectively.
