# Supplementary material for: Biaxial Tensile Strain-Induced Enhancement of Thermoelectric Efficiency of α-Phase Se2Te and SeTe2 Monolayers
Source: Nanomaterials (Basel). 2021 Dec 23;12(1):40. doi: 10.3390/nano12010040 (PMC8746480; doi:10.3390/nano12010040)
Supplement: Supplementary file 1 [file nanomaterials-12-00040-s001.zip › nanomaterials-1472567-supplementary.pdf]

nanomaterials

Supporting Information

Biaxial Tensile Strain-Induced Enhancement of Thermoelectric Efficiency of α-Phase Se_2Te and $SeTe_2$ Monolayers

Shao-Bo Chen [1,2], Gang Liu [3], Wan-Jun Yan [2], Cui-E Hu [4,*], Xiang-Rong Chen [1,*] and Hua-Yun Geng [5]

[11] College of Physics, Institute of Atomic and Molecular Physics, Sichuan University, Chengdu 610064, China; shaobochen@yeah.net
[2] College of Electronic and Information Engineering, Anshun University, Anshun 561000, China; yanwanjun7817@163.com
[3] School of Physics and Engineering, Henan University of Science and Technology, Luoyang 471023, China; liugang8105@haust.edu.cn
[4] College of Physics and Electronic Engineering, Chongqing Normal University, Chongqing 400047, China
[5] National Key Laboratory for Shock Wave and Detonation Physics Research, Institute of Fluid Physics, CAEP, Mianyang 621900, China; s102genghy@caep.cn
* Correspondence: cuiehu@cqnu.edu.cn (C.-E.H.); xrchen@scu.edu.cn (X.-R.C.)

Citation: Chen, S.-B.; Liu, G.; Yan, W.-J.; Hu, C.-E.; Chen, X.-R.; Geng, H.-Y. Biaxial Tensile Strain-Induced Enhancement of Thermoelectric Efficiency of α-Phase Se_2Te and $SeTe_2$ monolayers. *Nanomaterials* **2022**, *12*, 40. https://doi.org/10.3390/nano12010040

Academic Editor: Gyaneshwar P. Srivastava

Received: 4 November 2021
Accepted: 17 December 2021
Published: 23 December 2021

Publisher's Note: MDPI stays neutral with regard to jurisdictional claims in published maps and institutional affiliations.

Copyright: © 2021 by the authors. Licensee MDPI, Basel, Switzerland. This article is an open access article distributed under the terms and conditions of the Creative Commons Attribution (CC BY) license (http://creativecommons.org/licenses/by/4.0/).

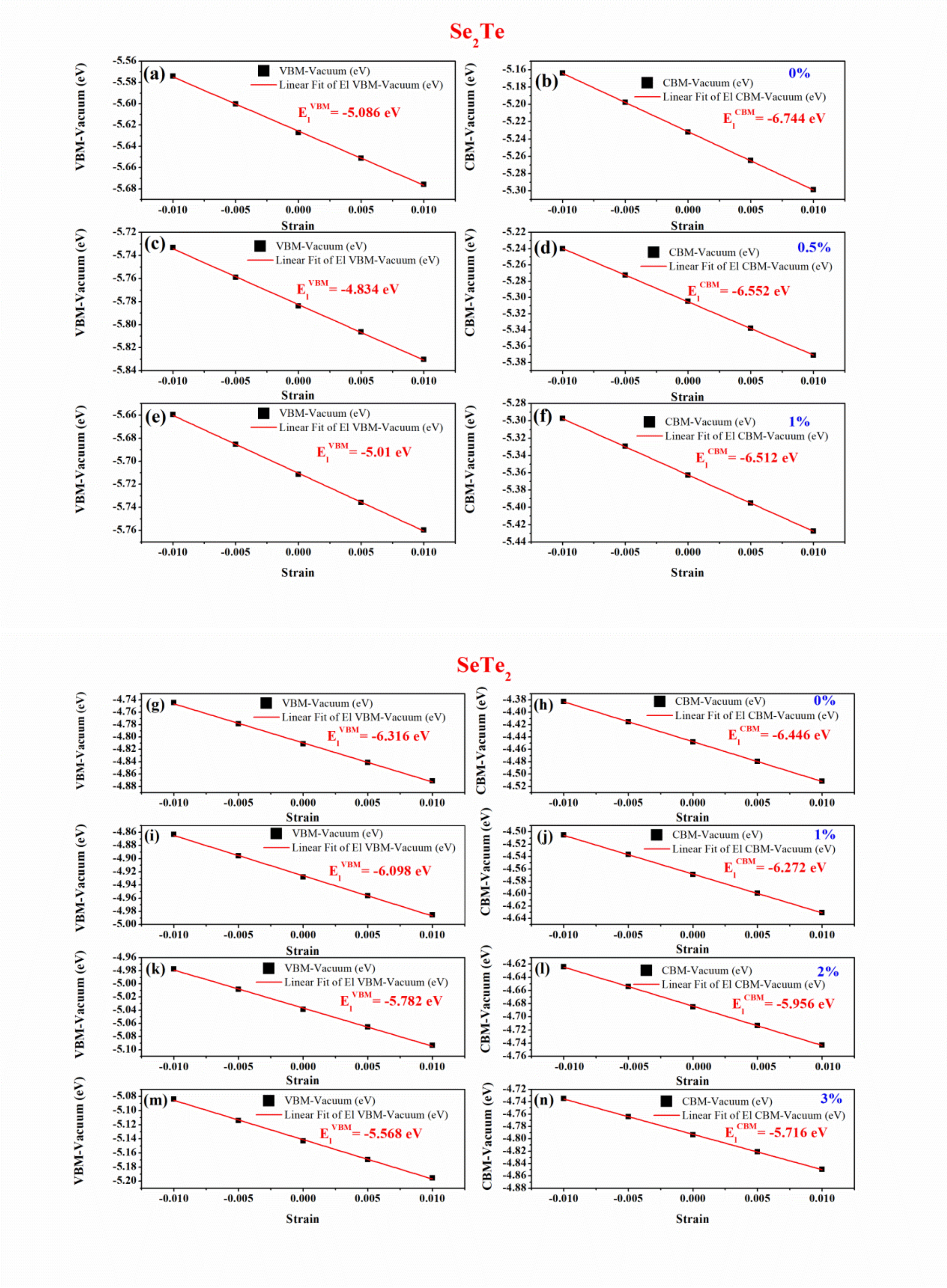

Figure S1. (Color online) The strain-dependent of deformation potential constants E_l of α-phase (a-f) Se_2Te and (g-n) $SeTe_2$ monolayer based on PBE+SOC band structures, respectively. The red straight line is the linear fitting curve.

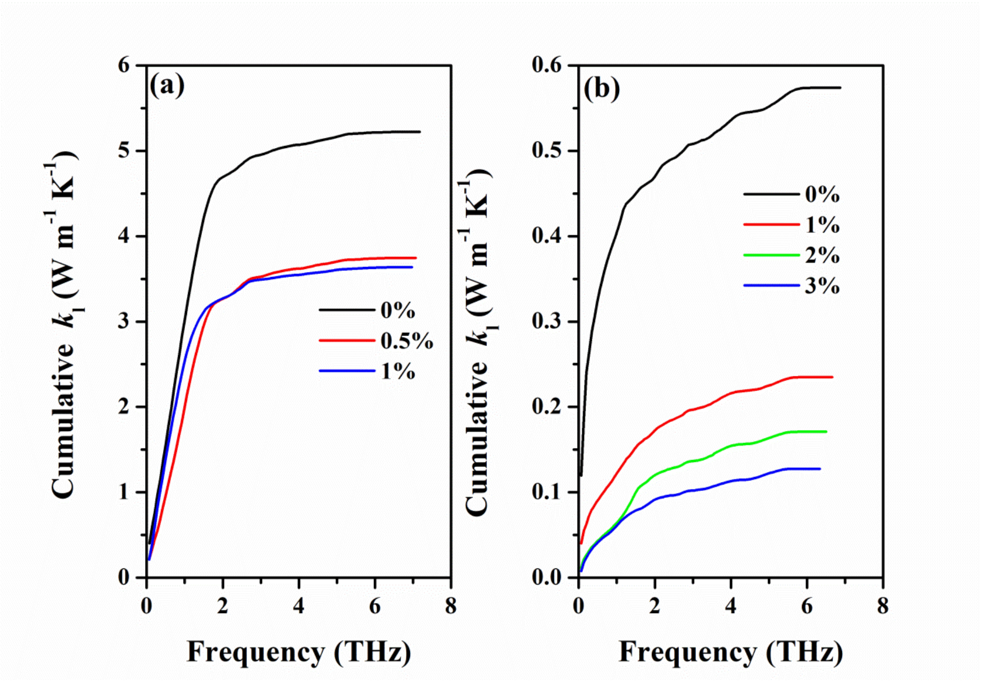

Figure S2. (Color online) The strain-dependent of cumulative k_l of (a) Se_2Te and (b) $SeTe_2$ monolayer at 300 K as a function of frequency, respectively.

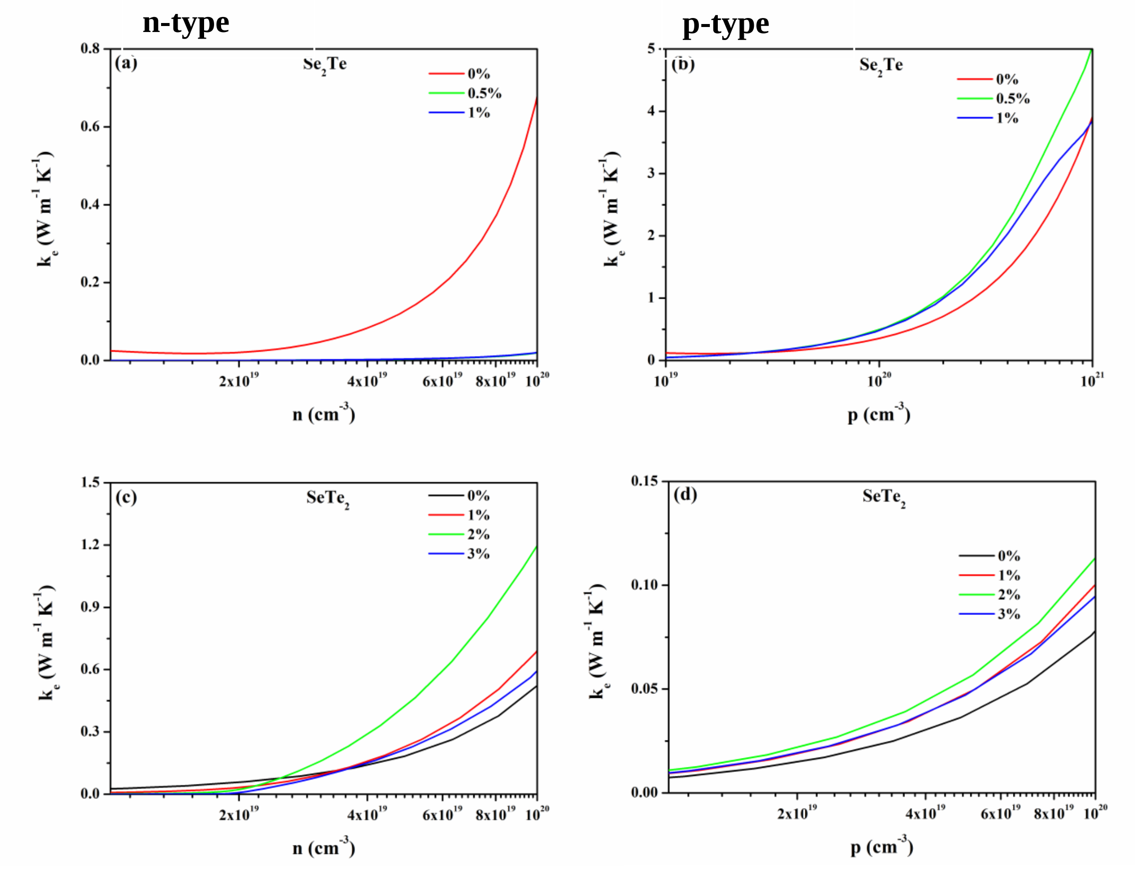

Figure S3. (Color online) The strain-dependent of electronic thermal conductivity k_e of (a, b) Se_2Te and (c, d) $SeTe_2$ monolayer at 300 K as a function of concentration for n-type and p-type doping, respectively.
